# Supplementary material for: Inhibition of PIP4Kγ ameliorates the pathological effects of mutant huntingtin protein
Source: eLife. 2017 Dec 26;6:e29123. doi: 10.7554/eLife.29123 (PMC5743427; doi:10.7554/eLife.29123)
Supplement: Source code 1. [file elife-29123-code1.docx]

function scores = newmachinecalcscores(trkname,config, cID)

% This function requires three inputs, trkname(with full path), config

% struct and cID(camera ID)

% Edo debug 61417

trkname = 'R10002_05_A08_2_1495753006.trk'

cfgname = 'SRImachine.cfg'

config = srireadconfig(cfgname);

cID=1;

% Read tracking data from file.

fid = fopen(trkname);

trkdata = fread(fid,[7 inf],'uint16')';

fclose(fid);

% Convert tracking file uint16 format to real format.

nrframes = double(trkdata(1,1));

imagewidth = double(trkdata(1,2));

imageheight = double(trkdata(1,3));

bigdim = imagewidth;

if imageheight > bigdim; bigdim = imageheight; end;

% Convert to real pixel numbers and radians

trajdata = [double(trkdata(:,1:2)) double(trkdata(:,3:6))*bigdim/65535 (double(trkdata(:,7)./65535-0.5)*2*pi)];

% Zero angle if angle is < eps

trajdata(find(abs(abs(trajdata(:,7))-pi)<eps),7) = 0;

% Remove Header

trajdata = trajdata(2:end,:);

% Find all the index of all starting tracks

trajstart = find(trajdata(:,1)==0); % Including zeroes row.

if length(trajstart)>0

trajlength = [diff(trajstart); size(trajdata,1)-trajstart(end)+1]; % Including zeroes row.

else

trajlength = [];

end

% Convert to camera position 1 coordinates.

trajnotstart = find(trajdata(:,1)>0);

cix = cID;

if cix == 2

xold = trajdata(trajnotstart,3);

xnew = (xold-config.bottom2)*((config.bottom1-config.grippertip1)/(config.bottom2-config.grippertip2))+config.bottom1;

trajdata(trajnotstart,3) = xnew;

end

if cix == 3

xold = trajdata(trajnotstart,3);

% xnew = (xold-620)*499/466+597;

xnew = (xold-config.bottom3)*((config.bottom1-config.grippertip1)/(config.bottom3-config.grippertip3))+config.bottom1;

trajdata(trajnotstart,3) = xnew;

end

if cix == 4

xold = trajdata(trajnotstart,3);

xnew = (xold-config.bottom4)*((config.bottom1-config.grippertip1)/(config.bottom4-config.grippertip4))+config.bottom1;

trajdata(trajnotstart,3) = xnew;

end

crossx = config.Cross_Lines./config.Downscale;

rlims = config.Frame_Rectangle./config.Downscale;

if rlims(3) <= 0; rlims(3) = imagewidth+rlims(3)-rlims(1); end;

if rlims(4) <= 0; rlims(4) = imageheight+rlims(4)-rlims(2); end;

rlims(3) = rlims(3) + rlims(1);

rlims(4) = rlims(4) + rlims(2);

scores = [];

xcoords = [];

speeds = [];

vspeeds = [];

turnings = [];

stumblings = [];

areas = [];

tcount = 0;

tlength = 0;

crosshigh = 0;

crosslow = 0;

fcounts = zeros(1,config.Nr_Frames);

% Loop through trajectories.

for tri = 1:length(trajstart)

% Single out current trajectory only.

currtraj = trajdata(trajstart(tri):(trajstart(tri)+trajlength(tri)-1),:);

% Find points that are within frame subset and rectangle.

firstframe = config.Frame_Subset(1);

lastframe = config.Frame_Subset(2);

if firstframe <= 0; firstframe = 1; end;

if lastframe <= 0; lastframe = config.Nr_Frames + lastframe; end;

valinds = find(currtraj(:,1)>=firstframe & currtraj(:,1)<=lastframe & ...

currtraj(:,3)>=rlims(1) & currtraj(:,3)<=rlims(3) & ...

currtraj(:,4)>=rlims(2) & currtraj(:,4)<=rlims(4) & ...

(currtraj(:,1)>15 | currtraj(:,3)>225)); % Remove errors from gripper (based on cam pos 1).

% Make sure this trajectory has at least the minimum length.

if length(valinds) >= config.Min_Trajectory_Length

% Exclude invalid frames.

currtraj = currtraj(valinds,:);

% Find leaps along the trajectory (if fly has passed out of rectangle and then back in).

% Also calculate x and y differences (movement) and exclude leap points.

leapind = find(diff(currtraj(:,1) ~= 1));

diffx = diff(currtraj(:,3));

diffx(leapind) = NaN;

diffy = diff(currtraj(:,4));

diffy(leapind) = NaN;

% Calculate scores. these grow for each iteration

xcoords = [xcoords; currtraj(:,3)];

speeds = [speeds; sqrt(diffx.^2+diffy.^2)];

vspeeds = [vspeeds; abs(diffx)];

directions = 180./pi.*atan2(diffy,diffx); %degrees

directions(find(directions>90)) = directions(find(directions>90)) - 180;

directions(find(directions<-90)) = directions(find(directions<-90)) + 180;

newturnings = abs(diff(directions));

newturnings(find(newturnings>90)) = 180 - newturnings(find(newturnings>90));

turnings = [turnings; newturnings];

if ~isempty(directions)

newstumblings = abs(directions - 180./pi.*currtraj(1:end-1,7));

newstumblings(find(newstumblings>90)) = 180 - newstumblings(find(newstumblings>90));

stumblings = [stumblings; newstumblings];

end

areas = [areas; currtraj(:,2)];

if any(currtraj(:,3)<crossx(1))

crosshigh = crosshigh + 1;

end

if any(currtraj(:,3)<crossx(2))

crosslow = crosslow + 1;

end

fcounts(currtraj(:,1)) = fcounts(currtraj(:,1)) + 1;

tcount = tcount + 1;

end

end

% Watch out for bad values. Remove all the NaN

xcoords = xcoords(find(isfinite(xcoords) & xcoords~=0));

speeds = speeds(find(isfinite(speeds) & speeds~=0));

vspeeds = vspeeds(find(isfinite(vspeeds) & vspeeds~=0));

turnings = turnings(find(isfinite(turnings) & turnings~=0));

stumblings = stumblings(find(isfinite(stumblings) & stumblings~=0));

areas = areas(find(isfinite(areas) & areas~=0));

tlength = sum(speeds);

% Make sure movie had enough trajectories.

if tcount < config.Min_Nr_of_Trajectories

scores = NaN*ones(1,12);

else

% The multiplications compensate for the effect of downscaling on scores.

scores = [config.Downscale*mean(xcoords) config.Downscale*mean(vspeeds) config.Downscale*mean(speeds) ...

mean(turnings) mean(stumblings) (config.Downscale.^2)*mean(areas) tcount length(xcoords) ...

config.Downscale*tlength crosshigh crosslow nanmax(fcounts)];

end
